# Care-managers’ professional choices: ethical dilemmas and conflicting expectations

**DOI:** 10.1186/s12913-017-2578-4

**Published:** 2017-09-07

**Authors:** Siri Tønnessen, Gøril Ursin, Berit Støre Brinchmann

**Affiliations:** 1 University College of Southeast Norway, Campus Vestfold, 3603 Kongsberg, Norway; 2 Nord University, 8049 Bodø, Norway

**Keywords:** Professional responsibility, Accountability, Care-managers, Ethical dilemmas, Qualitative research, Role expectations, Community services, Administrative decisions

## Abstract

**Background:**

Care-managers are responsible for the public administration of individual healthcare decisions and decide on the volume and content of community healthcare services given to a population. The purpose of this study was to investigate the conflicting expectations and ethical dilemmas these professionals encounter in their daily work with patients and to discuss the clinical implications of this.

**Methods:**

The study had a qualitative design. The data consisted of verbatim transcripts from 12 ethical reflection group meetings held in 2012 at a purchaser unit in a Norwegian city. The participants consist of healthcare professionals such as nurses, occupational therapists, physiotherapists and social workers. The analyses and interpretation were conducted according to a hermeneutic methodology. This study is part of a larger research project.

**Results:**

Two main themes emerged through the analyses: 1. Professional autonomy and loyalty, and related subthemes: loyalty to whom/what, overruling of decisions, trust and obligation to report. 2. Boundaries of involvement and subthemes: private or professional, care-manager or provider and accessibility.

**Conclusions:**

Underlying values and a model illustrating the dimensions of professional responsibility in the care-manager role are suggested. The study implies that when allocating services, healthcare professionals need to find a balance between responsibility and accountability in their role as care-managers.

## Introduction

Care-managers are professionals working in purchaser units in community healthcare. They are responsible for the public administration of individual decisions and deciding on the volume and content of care services allocated to a population [1]. As the need for nursing and healthcare generally exceeds available resources, the allocation of healthcare services often implies ethical dilemmas [2, 3]. To support healthcare professionals with difficult moral decisions they encounter in their daily practice, ethics reflection groups have been established in various clinical settings [4]. Even though there is a growing body of research connected to healthcare professionals taking part in ethics reflection groups [4–8], few studies have looked at the ethical dilemmas care-managers discuss as participants in such groups. The aim of this study, therefore, was to gain insights into the ethical dilemmas and conflicting moral expectations care-managers encounter in their daily work as they discuss these dilemmas in ethics reflection groups and to discuss the clinical implications of this.

## Background

As in many other Western countries, the Norwegian healthcare system is based on a liberal welfare model funded from the state budget [2]. The overall intention with regard to how public healthcare is organised is to ensure a just allocation and equal access to services as well as transparency in the decision-making process [9]. In the last decades, ideas from New Public Management have influenced the organisation of healthcare services in Norway [1]. In community healthcare, a purchaser-provider service (PPS) commonly aims to divide the responsibilities and duties of the care-manager purchaser and provider of services to clarify the role and power of the care-manager [10]. Hence, care-managers in the purchaser unit are public administrators and allocate services. Meanwhile, the health services to patients are provided by professionals working in a provider unit (called ‘providers’). This means that care-managers have a specific responsibility to allocate services fairly, which is often described in terms of a tension between individual good (particularity) and aggregated/collective good (utility/benefit) [11].

The introduction of a PPS is mainly intended to create competition between providers within the community [12] and establish quasi markets in the national health services [13] or, at least, marked reforms [14]. The establishment of PPSs within national health services is aimed at improving cost containment, efficiency and organisational flexibility and responsiveness to the services patients’ need [15]. There is little consensus on how care-manager functions should be formulated or organised to achieve these goals [13]. A general assumption, though, is that the care-manager is able to articulate the needs and wishes of the population and make plans for service delivery [15].

Contrary to the stated goals of the PPS organisational framework, research indicates that it actually leads to reduced flexibility regarding allocation time and in responding to the shifting demands of patients [3, 16]. In practice, providers and care-managers often cooperate closely to provide help in the best way possible to meet individual patient demands [17, 18]. Other studies indicate that care-providers use individual strategies that allow for flexibility and cooperation rather than rigid interpretation of laws and regulations [19–21]. Hence, this way of organising healthcare services – the introduction of PPSs – seems to disturb the provider-patient relationship and has implications for the quality of care and the idea of patients as unique persons who cannot be standardised as (if) mass products [22].

Research also highlights that care-managers transform politics into daily practice [23] and points to the tension between managing needs and risks, between user autonomy and protection [24], and the idea that ‘the rationale for decision making seemed to be more about what was defensible than what was right’ ([25]:1424). Meanwhile, care-managers transform the needs of elderly people into organisation services using their structural and intentional power [26] and adapting to the restrictive approach in relocation decisions rather than considering the needs of the patient [27].

Reviewing the literature on care-managers, several studies have argued that new public management (NPM) and care organisation using a PPS model represent an economic discourse [28–30], one that may lead to discrepancies between professionally accountable obligation and professional autonomy that negatively impact upon service quality [31]. Notably, accountability obligations may be more extensive than the degree of autonomy that professionals are permitted to exercise [32]. Furthermore, the care-managers make decisions that affect client outcome and overall system resources [33], while decision-making is influenced by the collective wisdom of the team, the role of family and the messiness of the process [34]. There is a tension and ambiguity that influences care-managers’ working conditions, thus contributing to increased stress levels and resultant staffing problems and affecting the quality of service given to older people in particular [35]. A study based on the experiences of care-managers reveals that they believed that relationships with patients have the greatest impact on patient satisfaction, while the support they provide clinicians has the greatest impact on clinician satisfaction [36]. Other literature describes how professional standards are being pushed to their limits [37] and how work-related stress has increased [38].

As mentioned, NPM implies a shift from an ethical to an economical discourse. This shift does not mean that ethical dilemmas are absent; rather, they are transformed and enacted within the practices of care-managers. This article highlights how care-managers struggle with ethical issues in a purchaser unit. Hence, the purpose of this article is to investigate the ethical challenges and dilemmas that professionals face in the provider office encounter while negotiating between economic, political and care values in their professional role. The role they play is defined as the set of behaviours, rights, norms and obligations regarding how professionals are supposed to act according to their position in the organisation, where role conflicts arise when these expectations are mutually exclusive [39]. Relatedly, in this article, ethical dilemmas involve care-managers having to make decisions but experiencing conflicting expectations as moral values conflict in a specific situation [11]. Moral values are taken to be values that derive from the significant moral intentions people have in upholding such things as human life, freedom, welfare, dignity, autonomy and justice. [11]. Through understanding the ethical dilemmas encountered by care managers, we can better focus on their decision-making practices to meet the diverse needs of patients and caregivers.

## Methods

### Study setting

This study is part of a larger ethics project initiated by the community healthcare sector in order to strengthen moral awareness among healthcare professionals employed in municipal healthcare services. As part of this project, ethics reflection groups were established in various clinical settings in municipal healthcare services in a city in Norway. A researcher (BSB) was contacted and invited to participate in the ethics reflection groups to conduct the related research [40–42].

Ethics reflection groups focus on supporting healthcare professionals in dealing with moral issues [4]. In the groups, the participants reflect on ethical issues from their daily work. The point of departure is concrete situations from the participants’ own clinical experience, and the focus of the reflection is on moral values and reasoning [43]. The group dialogue is facilitated by a trained facilitator (usually the head of the department or someone working in the clinical setting), fostering a structured reflection process. The aim of ethical reflection is to promote the participants’ moral reasoning and ethical awareness by reflective dialogue, and the objective is to ascertain the best possible solution to the dilemmas encountered [43].

In this article, we present some of the ethical challenges that emerged in the ethics reflection groups with care-managers within a purchaser unit.

### Design

The study had a qualitative design, as the objective was to understand how social experience is created and given meaning [44]. The underlying assumptions in this study were that reality can be interpreted in multiple ways and that understanding is dependent on subjective interpretation, the aim of the study and the questions asked [45]. In line with this perspective, data were analysed and interpreted according to a hermeneutic methodology as described in Kvale [45] and Kvale and Brinkmann [46].

#### Data collection and sample

Data consisted of 320 pages of verbatim transcripts from 12 group meetings with care-managers in the purchaser unit in the Norwegian city mentioned. The participating care-managers were healthcare professionals, such as nurses, occupational therapists, physiotherapists and social workers. The groups met twice monthly from April to December 2012, and each meeting lasted for up to 1½ hours.

A group leader in the purchaser unit led the discussions, which were systematically structured to help steer the issues discussed. Researchers from a university (BSB and GU) participated in the group meetings as observers, but their role changed somewhat over time. Sometimes the group leader challenged the researchers to take part in the conversation, especially if it had slowed down. For example, the leader might ask, ‘Do you have anything to say to this?’ or ‘Now we are a bit stuck, can you help us go further’? Thus, the group leader drew the researchers out of a purely observational role into a more participatory one.

The participants in the reflection group were informed verbally and in writing about the research project, and all participants signed an informational approval form (informed consent). The study was approved by the Norwegian Regional Committee for Medical and Health Research Ethics and registered by the Norwegian Centre for Research Data. All the data were anonymised and handled confidentially.

### Data analyses

The analyses were started by ST and BSB reading the transcriptions from each group reflection and noting which ethical issues or moral concerns the care-managers introduced in the sessions. Hence, we gained a sense of the whole, that is, of the various ethical issues that were most prominent in the 12 group discussions [45, 46]. Next, we read the text from each group meeting more thoroughly. While reading, quotations representing ethical challenges or conflicting expectations were underlined and different moral values were noted in the margin. For each group meeting, an overview was made of quotations representing different ethical challenges, conflicting expectations and the values involved. This is in line with Kvale’s [45, 46] first level of interpretation, aiming at understanding the care-managers self-understanding of the dilemma they encounter as reflected in each group meeting, through the eyes of the researchers.

In three of the 12 group discussions, the ethical issues concerned the working environment; therefore, since the focus of this study was on care-managers’ ethical dilemmas when allocating services in their encounters with patients, we chose to omit these. This left us with the data from nine group meetings. In these meetings, the participants raised dilemmas about how to handle different expectations and how to balance the various values at stake (e.g. autonomy and responsibility) that were relevant for this study.

The next level of interpretation was to take the ethical dilemmas – the conflicting expectations, moral values and their interrelatedness – to a higher level of abstraction. This entailed what Kvale calls ‘common sense’ and ‘theoretical interpretation’ [45, 46]; it involves a variety of interpretations, going back and forth and trying to comprehend the main picture of the data while holding true to the transcribed data from the reflection groups [44, 47]. Thus, we read the texts from the nine group meetings carefully again, interrogating the dilemmas, expectations and values in terms of the purpose of the study and ethics and asking ourselves ‘What is this really about?’ In this way, we were eventually able to identify subthemes and themes representing different dilemmas professionals in the purchaser unit seemed to encounter in their care-manager role. In five of the group discussions, the (two) subthemes that emerged related to the theme of *autonomy* and *loyalty*, and in the other four, the subtheme related to *boundaries of involvement*. Table 1 presents an overview of the two themes and related subthemes that emerged from the nine group discussions, which we elaborate on in the findings (below).

**Table 1:** Results

Theme	Subthemes	Conflicting expectations
Professional autonomy and loyalty	1. Loyal – to whom?	Patients` needs versus HHC capacity
	2. Decisions overruled	Autonomy in professional decision-making versus political intrusion
	3. Trust and obligation to notify	Client confidentiality versus obligation to notify
Boundaries of involvement	1. Private or professional	Patients` needs versus needs to be a private person in leisure time
	2. Purchaser or provider	Responsibility versus accountability in the role as care-managers
	3. Accessibility	Access for patients` - versus workload in the purchaser unit

### Strengths and weaknesses of the study

The data in this study was not originally intended as material for research purposes but was derived from ethics reflection groups initiated from clinical practice. This may be considered a strength, therefore, since the data collection was not steered by the researchers, so the participants were able to freely express the issues they wanted to discuss. The reflections were, however, facilitated by a group leader, which might have limited the participants’ openness and honesty in the discussion. Another weakness may be that the researchers became involved in the discussions and were expected to contribute with their ethical competence when the conversation ‘ran dry’, which may have influenced the material. Another possible weakness was that the researchers were not involved in posing in-depth questions, which could have enriched the material. At the same time, this can also be regarded as a strength because the informants were not interrupted and so could talk freely about what was important to them.

## Results

### Professional autonomy and loyalty

The theme of professional autonomy and loyalty concerns the challenges faced by informants in making independent, sound and diligent decisions based on their professional judgement. The first two subthemes identified relate to making administrative decisions; *who/what to be loyal to when allocating services, the patient or limited resources*, and c*onfusion when decisions made are overruled*. The third subtheme relates to the *balance between trust and obligation to report*.

### Loyal – To whom/what?

In the group meetings, issues about who or what to be loyal to when allocating services was a frequently discussed theme. The participants described how they are torn between the patient’s need for nursing care and the pressure to allocate at a minimum level due to limited resources. As one group member said:

*As care-manager, I struggle to meet expectations from involved parties. We are under great pressure due to limited resources; we grant too many administrative decisions and too much time. They expect us to spare Home Healthcare (HHC). It’s a compound pressure. If there were enough nursing homes, patients would have been placed there. Instead, we increase the time granted in HHC services, but we know that HHC doesn’t have these resources. We are squeezed; it’s an enormous pressure.*

The participants said that they are obliged to base administrative decisions on the patients’ needs, but the pressure for minimum usage of recourses makes them unsure about what to measure, ‘the patients’ needs for services or the capacity in HHC to provide these services’. Either way, they describe challenges. If they grant services according to patients’ needs, next of kin call and make claims because the HHC does not have the capacity to provide services according to the decision. As one participant said:

*Then, next of kin call and say, ‘She was promised this and that care, but this is not what she’s getting.’ I feel like we’re ‘playing an office’ as to what our responsibility is. All the time, you’re stuck between a rock and a hard place. How are we supposed to be taken seriously? This is a major problem.*

Participants described how the construction of services is complex at several levels. In order for the healthcare services to be considered reliable among patients and their families, they have to match with needs, grant services and performed services. Sometimes professionals at the provider office have to grant services according to the capacity in HHC rather than in line with their professional judgments. This is particularly problematic, as they have to defend a decision that is not legitimate. As one participant said:

*We are supposed to assure the patient and their next of kin that the service is sound and diligent, adequate and sufficient. […] What can I say? Who to be loyal too? Should I say I disagree with the decision? I’m supposed to be loyal to both the patient and the workplace. I can’t say what I mean, but I can’t defend the decision either.*

Furthermore, they have to prioritise between patients’ needs and fiscal constraints, which they describe as political, rather than executive, which makes them question the connections between responsibility, loyalty and transparency within the system:

*What is loyalty without responsibility? And what are we responsible for? A sound and diligent service and to notify if the service is inadequate and insufficient. […] One needs to fix what isn’t working. Isn’t that a part of the concept of responsibility? […] Responsibility and transparency aren’t contradictions. What I experience now is the responsibility is to shut up and keep your cards close to your chest, and you are irresponsible if you open up. I think this is totally wrong.*

### Decisions overruled

The participants also described how politicians and chief municipal executives sometimes give in after pressure from next of kin and impose changes to administrative decisions on care-managers. Hence, their professional judgement in the role of care-manager is overruled. One participant said:

*Next of kin sent emails here, there and everywhere, all the way to the top. […] In the end, the whole municipality was involved. […] The mayor didn’t want bad publicity about the case, so he came and contacted the manager of the purchaser unit and our administrative decision was overruled.*

In this situation, the participants faced compound pressure from the mayor and deputy mayor, and experienced their professional judgement as less important than following political instructions. This was described as confusing, causing them to question their own judgement and responsibility. They also wondered if politicians can rightfully overrule them; pertinently, they felt, their decisions were sound and diligent professional judgements, and legitimate changes in administrative decisions should only be made when based on changes in the patient’s situation, not due to pressure from next of kin or politicians.

At the same time, participants explained how similar situations had been resolved differently by the former mayor. This indicates that the political establishment does indeed have an impact on the work care-managers do in the PPS. The care-managers said that politicians and administrative superiors should respect their professional judgement.

### Trust and obligation to notify

Participants reported that a part of their responsibility is to allocate services to people in vulnerable situations, such as young parents with drug problems. In some situations, they gain insights into people’s private lives and receive in-depth information about difficult life conditions. Hence, care-managers struggle to balance client confidentiality and the obligation to notify, for example, in order to protect children from unhealthy living conditions, even though these patients tend to ‘pour their hearts out’ to them. Moreover, care-managers at the purchaser units gain the trust of their clients, which creates further issues:

*Our clients say, ‘I thought you were here to help me, but you didn’t. When people ask for help, and then you experience that what you say is brought forward [to the authorities], it feels like betrayal, like going behind our backs.’ We do say that, according to the law, we have to put forward information, but the law is one thing and how it feels is another.*

Whether or not to diffuse information is a professional judgment, one that is important in terms of the perception of a trusting relationship; decisions on matters of this sort can be a time-consuming activity.

### Boundaries of involvement

The participants often discussed ethical challenges concerning the boundaries of involvement and the difficulty of setting limits for themselves in their roles as care-managers. The subthemes here were *balancing the private and professional*, *being a care-manager or provider*, and *being accessible*.

### Private or professional?

In one group meeting, the discussion revolved around how the participants set limits in their private lives regarding their professional roles as care-managers. This challenge presents itself when patients or next of kin want to be friends on social media, such as Facebook, or when they call the care-manager at home. One participant said:

*If I am personable and pleasant in the encounter with next of kin, they misunderstand and send friend requests on Facebook. […] Facebook is private, but it’s difficult to ignore friend requests. Home is private, and I don’t want to drag work home.*

The care-managers said that it is difficult to ignore friend requests, even though they do not have a relationship based on friendship as such. One participant said that she accepted a patient as friend on Facebook, and later she was asked on a date. Several participants suggested that official guidelines setting boundaries for relationships between professionals and patients might be helpful. However, they also said they understood that sometimes the patient and next of kin can have a desperate need to contact them.

Similarly, the care-managers explained that it is difficult to be professional if they become too emotionally involved. Hence, they try to avoid being care-managers for neighbours, friends or other acquaintances. As one group member said, ‘If I am in doubt, I always ask the patient if it’s ok for them that I’m managing their case’.

### Care-manager or provider?

In some situations, care-managers find it difficult to find the right balance between being a care-manager and being a provider. This dilemma arises mainly when they enter the patient’s home to map care needs. As one group member said:

*Sometimes it’s difficult just to stick to the care-manager role. It’s is unnatural to distinguish administrative decisions from provision of care. It’s an artificial distinction. Caring is about trust, how to reach out to patients and the patient’s request and need for care in the situation here and now.*

In one case, the home of a patient was extremely messy and dirty, but the patient did not want any help. The care-manager was a trained nurse, her experience guided her to exceed the expectations in her role as care-manager and she became a provider. Moreover, she claimed, practical work was necessary for patients to accept help from the healthcare system:

*I’ve taken out garbage and cleaned the apartment and given the patient a bath and a manicure to gain entry so they can accept help from HHC. It is easier [for them] to trust the service if you make an effort and show them you care.*

Participants also described how the PPS model is too detailed to be useful because many providers only relate to the administrative decisions in a situation, so flexibility in the encounter with the patient vanishes. The administrative decisions limit the possibility of providing sound and diligent services in the real life context. As one care-manager said:

*We’ve disfranchised judgement and flexibility from the provider organisation. The PPS organisation fails as it’s steered in detail, so the providers can’t think for themselves. If the administrative decision doesn’t say they should dust, they don’t dust, but if it says dust, they might not take out the garbage.*

### Accessibility

Another dilemma concerns accessibility. As one group member said, it is difficult to ensure easy access to services and at the same time run the purchaser office efficiently:

*We need to introduce some boundaries to limit access to the care-managers, but at the same time secure accessibility for those who need necessary healthcare services from the purchaser office.*

The care-managers discussed using information technology (IT) to reduce the workload, but they worried that this would limit access for vulnerable patients:

*When you’re sick, you’re vulnerable, and especially the elderly might have problems using modern technology, and most of our patients are elderly. Access to the purchaser unit is relatively easy today; people only have to make a call or send an email.*

The participants claim that introducing modern technology might be efficient for them in the purchaser unit; however, it might be time consuming and a disadvantage for many elderly patients.

## Discussion

The main findings in this study, as presented in Table 1, concern how the care-managers struggle to find the balance between professional autonomy and loyalty and how they deal with setting boundaries of involvement in their professional roles. This might imply that they struggle to find the balance between particularity and utility/benefit in the situation and find it difficult to come to terms with and/or are uncertain about exactly what their responsibilities are in their role as care-managers. In respect of the informants’ efforts to balance utility/benefit and particularity, the findings illustrate that they also struggle to balance distance and closeness in their encounters with patients and next of kin. This can be interpreted as role confusion at both an individual and organisational level, as they meet contradicting expectations when values conflict. In Table 2, we have tried to capture the values in conflict related to the individual and organisational levels based on the themes and subthemes.

**Table 2:** Themes, subthemes and conflicting values

Subthemes	Loyal – to whom?	Decisions overruled	Trust and obligation to notify	Private or professional	Care-manager or provider	Accessibility
Themes	Professional autonomy and loyalty			Boundaries of involvement		
Conflicting values	Institutional level: Particularity - Utility/Benefit					
	Individual level: Closeness - Distance					

According to PPS, a primary expectation in the care-manager role is that resources be allocated fairly [15]. However, resources are limited, which implies a focus on utility and benefit [11]; at the same time, as patients, at least in Norway, they can legally claim services on an individual basis [48]. This dilemma between utility/benefit and particularity emerges as the consequence of an inherited dilemma derived from policymakers who decide what patients can claim through legislation, allocation of resources and the chosen organisation of healthcare services, which is expressed in municipalities as a capacity problem [3]. This dilemma is only expected to increase as medical possibilities to offer treatment continue to rise and more people need healthcare services as the population ages [49], medicalisation increases [50] and resources remain limited. Thus, it is argued that policymakers and politicians need to make changes in legislation and how healthcare is organised specifically to meet these challenges [3, 51, 52].

In the meantime, it is of the upmost importance that care-managers facing these dilemmas in their daily work gain insights that might help them make the best decisions possible. Our experience as nurse educators and researchers is that most nursing students start their education with a genuine desire to help and do good for people in need. These good intentions are elaborated further during their education as the fundamentals of nursing care, namely, to promote health, prevent illness, restore health and alleviate suffering in such a way that patients are cared for in a respectful, sound and diligent manner [53].

However, research indicates that students and newly graduated nurses are surprised by the discrepancy between the ideals gained through nursing education and the task orientation of clinical practice [54]. Newly graduated nurses say they want to do a good job and that they will quit their jobs if they have to compromise their professional integrity and feel they are not able to do good for the patient [55]. Studies indicate, however, that nurses and other healthcare professionals experience moral frustration and distress; that is, they know what the right thing to do is but are prohibited by organisational constraints, such as staff shortages, to act accordingly [35, 56, 57].

Moral distress has been widely discussed in the nursing literature [58, 59]. In the classic article by Corley [57], this is mainly discussed at an individual level while emphasising the need for further development on the organisational/institutional level as moral distress is caused by institutional constraints. Hence, we found it interesting to try to capture how these two levels might be connected as related to the professional responsibility and sense of conflicting demands and unmet needs described in the findings in our study.

### Dimensions of professional responsibility

**Fig. 1:**
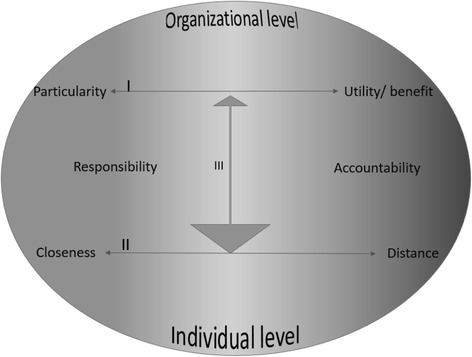
Dimensions of professional responsibility

In our effort to interpret and understand the findings illuminated in this study, we present a model for analytical purposes that suggests the connections between various dimensions of professional responsibility and the care-manager role (see Fig. 1).

Here, we make a distinction between the individual and organisational levels, as represented by the two horizontal arrows. At the individual level, care-managers balance between closeness and distance, while at the organisational level, they weigh up issues related to particularity as against utility/benefit. In real life, however, these two levels very much influence each other, as shown by the vertical arrow. According to the informants in our study, the organisational level influences the individual level more than the reverse, hence the different sizes on the arrows ends. In the theoretical discourse, ethical issues, such as closeness and distance as related to professional responsibility and particularity and utility/benefit as related to justice, are often presented as dichotomies. We have chosen to present these issues in the model rather as continua, however, and not as either/or, as a continuum seems more in line with the experiences of the informants when describing their clinical practice. Hence, in the care-managers experience struggles in trying to find a balance – the middle ground – between the extremes on the continuum when making their professional judgments [34, 37]. This is particularly the case where organisational limits are prominent.

On the left side, between the horizontal arrows, we have placed the concept of responsibility, and on the right side, accountability. Responsibility and accountability are two dimensions emphasising different values and expectations about what care-managers are responsible for. As they move along the continuum, they go towards particularity and closeness, values related to the traditional concept of responsibility as professional conduct [31, 60, 61]. In nursing, responsibility as a concept is closely linked to ethics and the expectations that the nurse voluntarily involves their capacity to act in a morally responsible way for the best interest of the patient [53, 62].

Accountability is related to the practice of accounting and associated with individualism, competition, control and efficiency [60, 61]; hence, we see the move towards distance and utility/benefit in the illustration. As public services draw heavily on ideas from NPM, such as PPS, it is argued that professionals need to bring responsibility ‘back in,’ since accountability is something of a contradiction in terms in professional practice [60, 61]. For the care- managers, it is evident that this balance is particularly difficult insofar as they are supposed to make professional judgements that rest on the values and expectations of their professional obligations as, for example, nurses, and the mandate and authority they hold in their managerial role as care-managers in the organisation.

These findings illustrate how difficult it is to merge the different expectations in the care-manager role. It seems, indeed, that care-managers rest their professional judgments at different places on the continuum depending on which values they emphasise in any one encounter with patients and how much pressure there is from the values and expectations representing the organisational level. Further, the continuum illustrates a main point, that both the patients and professionals are unique individuals who may place themselves at different positions on the continuum. Hence, it is important in a professional team that one can fill the continuum with different people to meet the patients’ individual needs. However, the extremes involve pitfalls at both levels.

### Implications for practice

The implications for practice at both individual and organisational levels may be understood as involving discussion of this continuum as a tool to develop and understand the care-manager role more thoroughly. At the individual level, it is especially important to consider the balance between closeness and distance in order to remain professional, that is, to keep some distance to maintain an overview while at the same time being sensitive to the patients’ individual needs. The findings indicate two pitfalls in this respect: being too distant and being too private. The need for closeness and distance will vary as patients’ and their needs differ, but the question of how close or how distant one can or should be as a professional while meeting the patients individual needs in a sound and diligent matter is the balance that care-managers need to find. Therefore, it is important that they do not lose their good intentions, which is decisive for moral sensitivity in encounters with patients [63]. However, the findings indicate that they need to protect themselves from becoming too involved; for example, the time when they are not at work is private time. To uphold a level of private autonomy–to find, that is, limits (boundaries) that help them distinguish between the different roles–seems to be important.

At an organisational level, there is a need to discuss the balance between particularity and utility/benefit, especially at the policy level, as it is the care-manager role to make decisions about budgets and organisational structure. It is evident that PPS not is in line with professional values, and the care-managers are prominent examples of how difficult it is to merge conflicting expectations stemming from a combination of two ideologies in one role. There is also, as indicated, a need for clarification of the care-managers’ responsibility when decisions are overruled. This is an infringement of professional autonomy, and the legitimacy of such interference ought to be discussed according to the relevant legislation.

## Conclusions

In this article, we have investigated how autonomy and loyalty and the boundaries of involvement are themes representing conflicting expectations in the care-manager role. The findings indicate that care-managers balance the opposing values of closeness and distance, on the one hand, and particularity and utility/benefit, on the other, and that these represent pitfalls at the extremes of two continua, the individual and organisational levels. Further, we have presented a model suggesting connections between these different dimensions of professional responsibility in the care-manager role, emphasising the distinction between the concepts of responsibility and accountability and discussing related implications for practice.

As far as we know, few researchers have captured the dimensions of professional responsibility as people in the care-manager role balance between clinical and managerial obligations. Hence, the findings are both relevant and important for care-managers as a point of departure for reflection and decision-making as well as for ethicists, clinicians and policymakers.

## Abbreviations

HHC: Home healthcare
NPM: New public management
PPS: Purchaser-provider service
